# The Concentrations of Phenolic Compounds and Vitamin C in Japanese Quince (*Chaenomeles japonica*) Preserves

**DOI:** 10.3390/foods14081369

**Published:** 2025-04-16

**Authors:** Renata Kazimierczak, Klaudia Kopczyńska, Alicja Ponder, Ewelina Hallmann, Małgorzata Żebrowska-Krasuska, Dominika Średnicka-Tober

**Affiliations:** 1 Department of Functional and Organic Food, Institute of Human Nutrition Sciences, Warsaw University of Life Sciences, Nowoursynowska 159c, 02-776 Warsaw, Poland; renata_kazimierczak@sggw.edu.pl (R.K.); klaudia_kopczynska@sggw.edu.pl (K.K.); alicja_ponder@sggw.edu.pl (A.P.); ewelina_hallmann@sggw.edu.pl (E.H.); malgorzata_zebrowska@sggw.edu.pl (M.Ż.-K.); 2 Bioeconomy Research Institute, Agriculture Academy, Vytautas Magnus University, Donelaicio 58, 44248 Kaunas, Lithuania

**Keywords:** Japanese quince, preserves, bioactive compounds, phenolic acids, flavonoids, vitamin C, alternative sweeteners, honey, cane sugar, xylitol, white sugar

## Abstract

The aim of this study is to characterize a range of the Japanese quince (*Chaenomeles japonica*) fruit preserves in terms of the content of vitamin C and phenolic compounds, as well as to discuss the effects of processing on the concentrations of these compounds in Japanese quince fruit. Research materials consisted of seven different products: a 100% Japanese quince fruit pressed juice, syrups with added honey, cane sugar, and xylitol, and three products sweetened with white sugar: jam, fruits in syrup, and candied fruits. The content of vitamin C and polyphenolic compounds (phenolic acids and flavonoids) was determined by high-performance liquid chromatographic method. The study confirmed that the Japanese quince fruit preserves can be considered a rich source of vitamin C and selected phenolics. At the same time, the tested products differed significantly in terms of the levels of the analyzed phenolic compounds, e.g., the syrups sweetened with xylitol were characterized by significantly higher concentrations of the analyzed phenolics than the syrups sweetened with other sweeteners. It is noteworthy to explore the possibilities for quince fruit processing, taking into account various processing methods and conditions, and using sweeteners alternative to white sugar.

## 1. Introduction

In recent years, much attention has been paid to the content of bioactive compounds with antioxidant properties in various crops, mainly due to their importance in preventing many non-communicable diseases. Multiple scientific studies have shown that there is a significant link between the consumption of these natural antioxidants and a reduced incidence of, i.e., heart diseases, cancers, and other degenerative diseases [1,2]. The protective role of fruits and vegetables against these diseases is attributed to several groups of antioxidants, including polyphenols and vitamin C. Fruits of various species, including *Chaenomeles*, are recognized as a rich natural source of these antioxidants [3,4,5,6,7,8]. The *Chaenomeles* fruits (*Rosaceae* family, *Pomoideae* subfamily), called “Japanese quince” or “quince”, were known in China thousands of years ago, but they have been cultivated in Europe since the end of the 19th century. Interest in quince increased one century later due to the favorable growing conditions in Europe. Fruits of this species are irregular, apple-shaped, and usually reach about 50 g of weight. The flesh is light yellow and hard and is characterized by a high content of essential oils, making the ripe fruit very aromatic [9]. Japanese quince fruit is rich in bioactive compounds that offer various health benefits. Key compounds include phenolics, such as derivatives of quercitrin and rutin, catechins, and chlorogenic acid, which possess strong antioxidant and antimicrobial properties [10,11], as well as vitamin C, acting as a potent antioxidant and contributing to chronic disease prevention [10,12]. The dietary fiber contained in fruits also plays an important role in supporting digestive health and aiding in managing body weight and diabetes [10,11], while triterpenes (oleanolic acid and ursolic acid) are responsible for anti-inflammatory, anticancer and antihyperlipidemic effects [8]. These compounds collectively enhance immune function, reduce inflammation, and may have protective effects against certain diseases. The concentration of vitamin C and polyphenols in Japanese quince can vary based on factors such as genotype, environmental conditions, and processing methods.

Although Japanese quince fruits are rich in antioxidants, they are not popular in direct consumption because of their high bitterness and tart taste. Therefore, methods of quince processing have developed intensively in recent decades. Furthermore, researchers have demonstrated the possibility of using quince processing by-products (seeds, leaves and pomace) as a source of bioactive compounds, mucilage, and fibers used in the pharmaceutical and cosmetic industries as well as in the production of bio-based films [10,13,14,15].

Due to the health-promoting effects of antioxidants, their content in food is one of the factors influencing consumers’ dietary choices. At the same time, it is known that food processing significantly affects antioxidants, such as polyphenols, through various mechanisms, which can alter their chemical structure, bioavailability, and biological activity. The technological processes can either enhance or degrade the compounds in foods and increase or limit their bioavailability depending on the products’ composition and conditions applied [16,17]. High temperature during processing may lead to polyphenol degradation and polymerization. At the same time, frying can increase certain polyphenols concentrations (e.g., hydroxycinnamic acids) due to isomerization and hydrolysis reactions. Moreover, polyphenols interact with carbonyl compounds during Maillard reactions, forming adducts that may inhibit harmful compounds like advanced glycation end-products. Binding of polyphenols to proteins or polysaccharides during heat treatment may also lead to their increased thermal stability and antioxidant activity [18]. On the other hand, non-thermal processing can improve the retention and bioavailability of polyphenolic compounds, due to, i.e., disruption of polysaccharide bonds in cell walls and membranes, inhibition of enzyme activity, free radical reactions, plant responses to stress, and interactions of polyphenols with the food matrix [19].

The fruits of *Chaenomeles* are commonly used in industrial processing [14]. They are mostly processed into juices, jams, liquors, purées, smoothies, and candied fruits. Moreover, they are added to teas, yogurts, lemonades, ice cream, cottage cheese, and confectionery, enriching these products with antioxidants. Japanese quince fruit juice can be useful as an acidulant with high antioxidant properties [20,21]. Strong competition and consumers’ expectations force producers to create products with high sensory attractiveness and health-promoting properties. Due to the high content of organic acids, the juice of Japanese quince fruits is very sour and thus does not reach high consumer acceptance if consumed directly, but it can be used as an additive to other products. Its sensory attractiveness may also be improved with the addition of various sweeteners. Since white sugar, commonly used in fruit preserves, is not recommended for frequent consumption, it is often replaced with other sweeteners, such as honey, cane sugar, or xylitol [22,23]. These sweeteners, in addition to having a significant impact on the sensory characteristics of products and their physicochemical properties, also show a health-promoting potential. For many consumers, this is an important aspect influencing their choice of such products [24].

The quality of Japanese quince (*Chaenomeles japonica*) fruit products is a relatively new subject of research. To date, very few studies can be found comparing the concentration of biologically active substances in their various preserves. Despite its rich nutritional composition and health-promoting potential, this fruit is not appreciated due to its pulp hardness, bitterness, and astringency [25]. Therefore, it seems appropriate to determine whether Japanese quince products processed towards reaching a higher sensory attractiveness are still characterized by a high content of biologically active compounds and, thus, what role they can play in health promotion. The aim of this study was, therefore, to characterize a range of Japanese quince (*Chaenomeles japonica*) fruit preserves in terms of the content of vitamin C and phenolic compounds, as well as to discuss the effects of processing methods and type of sweeteners on the concentrations of these compounds in the Japanese quince fruit.

## 2. Materials and Methods

### 2.1. Research Material

The analyzed material included seven processed Japanese quince products: pressed juice, syrup with honey, syrup with cane sugar, syrup with xylitol, and three products sweetened with white sugar: jam, fruit in syrup, and candied fruit. All Japanese quince samples came from the on-farm processing of the Japanese quince farm, located in Grajewo, Podlaskie Voivodeship in the eastern part of Poland (53°38′48″ N 22°27′18″ E). All of the preserves tested in this study are currently available for sale to consumers. The fruits used for the production of preserves belonged to one variety and were grown in the same soil and climate conditions. The fruits came from the harvest in 2019 and were processed immediately after harvest.

All recipes for the studied processed products were developed by the producer. For the pressed juice production, fresh quince fruits were washed and dried by fresh air at room temperature (21 °C). After drying, Baesso F20 Fruit Press was used for fruit squeezing and juice separation. The obtained quince juice was poured into glass bottles.

For the candied fruit production, fresh quince fruits, after washing, were cut into slices, and the seeds were removed. Next, fruit slices were put into glass jars and covered with sugar in a 1:1 ratio. The jars were kept for 3 days at room temperature until the sugar dissolved completely. The next step was to drain the resulting syrup and dry the fruit pieces in the oven (Binder Avantgarde Line, series FED-720) at a temperature of 55 °C, with airflow, for 3 days.

The syrups were obtained in the above-described procedure of candied fruit production, either with cane sugar (in a 1:1 ratio), linden honey (in the proportion of 0.6 kg of honey per 1 kg of quince fruit), or xylitol (in the proportion of 0.25 kg of xylitol per 1 kg of quince fruit). The effluent syrup was poured into glass bottles and closed. To prepare the fruits in syrup, fresh quince fruits were washed, peeled, seeded, cut into slices, and placed in 2-L jars with sugar in a 1:1 ratio (fruit slices were thoroughly coated with sugar) for 3 days. For quince jam production, fresh quince fruits were washed and cut into quarters. The seeds were removed, and the fruit quarters were smashed in a blender. The pulp was cooked in a pot for 20 min until the juice was released. Sugar was added in a 1:0.5 ratio, and the cooking was continued until a brown-red color and firm, dense consistency were reached. The preserves (3 technological replications of each of the product types) were provided for analysis to the Department of Functional and Organic Food at the Warsaw University of Life Sciences (Poland).

### 2.2. Chemical Analyses

#### 2.2.1. Dry Matter

The content of dry matter in the tested samples of quince products was determined by a weight method according to the Polish Standard (PN-R-04013:1988) [26] as previously described by Kazimierczak et al. [27]. The method principle is to dry the samples until constant weight, under specified conditions. Empty glass beakers were weighed, filled with quince products, and weighed again. Then, samples were dried at 105 °C, 1013 hPa, with free air circulation, for 72 h in an FP-25W Farma Play dryer (Poland). Then, samples were cooled to 21 °C and weighed again. The dry matter content was calculated (in g/100 g fresh weight) based on mass difference before and after the process of drying.

#### 2.2.2. Vitamin C

The concentration of vitamin C in the quince products was determined by the High-Performance Liquid Chromatography (HPLC) method, as previously described by Kazimierczak et al. [28], using Shimadzu equipment (USA Manufacturing Inc., Canby, OR, USA) with two LC-20AD pumps, a SIL-20AC autosampler, a CMB-20A system controller, a CTD-20AC oven, an ultraviolet-visible SPD-20AV detector, and a Hydro-RP 80A column (250 mm × 4.60 mm, particle size: 4 µm). Fresh samples of quince preserves (5 g of candied fruits, fruits in syrup, jam, or 5 mL of fresh quince juice and syrups) were extracted with 5% metaphosphoric acid in falcon testing tubes (10 mL), then mixed by Micro-Shaker 326M (Marki, Poland), incubated in an ultrasonic bath for 15 min, at 5 °C (5500 Hz), and then centrifuged (6000 rpm, 10 min, 0 °C). The supernatant (100 µL) was injected onto the Phenomenex Hydro 80-A RP column (250 × 4.6 mm). The analysis parameters were as follows: mobile phase of 50 mM acetic buffer (pH 4.4) prepared with 0.1 mM sodium acetate and 0.1 mM acetic acid (flow rate 0.5 mL min^−1^), analysis time: 18 min and detection wavelength: 255–260 nm. Identification of individual compounds: L-ascorbic acid (L-Asc) and dehydroascorbic acid (DHA) was based on Fluka and Sigma–Aldrich (Warsaw, Poland) standards with 99% purity.

#### 2.2.3. Phenolic Compounds Extraction and Identification

**Figure 1:**
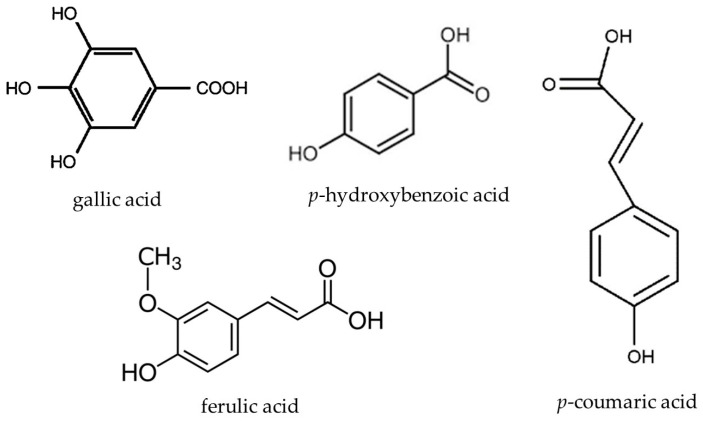
Structure formulas of the quantified phenolic acids.

**Figure 2:**
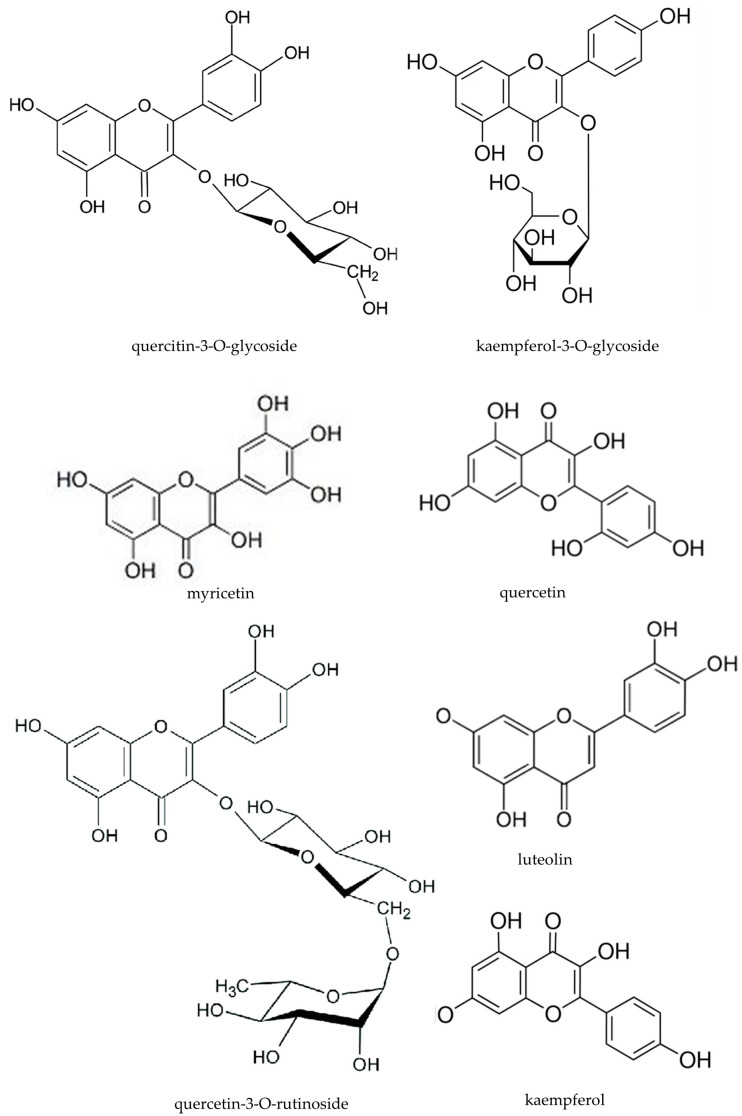
Structure formulas of the quantified flavonoids.

The content of phenolic acids and flavonoids in quince product samples was analyzed by High-Performance Liquid Chromatography (HPLC), as previously described by Kazimierczak et al. [28]. The analyses were conducted with the use of the Shimadzu HPLC equipment, as characterized in the previous manuscript section. Fresh samples of quince preserves (1 g of candied fruits, fruits in syrup, jam, or 1 mL of fresh quince juice and syrups) were mixed with 80% methanol (10 mL), with the use of a Micro-Shaker 326M (Poland), and incubated in an ultrasonic bath for 10 min at 30 °C (5500 Hz). The sample was then centrifuged (3450× *g*, 12 min, 2 °C) and the 1 mL aliquots of supernatant were injected onto the HPLC Synergi Fusion-RP 80i Phenomenex column (250 mm × 4.60 mm). The time of analysis was 38 min. Polyphenols were separated under gradient conditions with a flow rate of 1 mL min^−1^ by applying an aqueous solution of 10% (*v*/*v*) acetonitrile (phase A) and 55% (*v*/*v*) acetonitrile (phase B), both acidified by ortho-phosphoric acid to pH 3.0. The phases changed as follows: 0–21.00 min: 95% Solvent A and 5% Solvent B; 21.01–25.00 min: 50% Solvent A and 50% Solvent B; 25.01–27.00 min: 20% Solvent A and 80% Solvent B; 27.01–32.00 min: 20% Solvent A and 80% Solvent B; and 32.01–36.00 min: 95% Solvent A and 5% Solvent B, with the flow rate of 1 mL min^−1^. The wavelengths used for detection were: 370 nm for flavonols, and 250 nm for phenolic acids. For individual phenolic acids and flavonoids identification, the Sigma–Aldrich (Warsaw, Poland) external standards with HPLC purities of 95.00–99.99% were used. Figure 1 and Figure 2 present the structure formulas of all the quantified phenolic acids and flavonoids, respectively.

The concentration of compounds was calculated based on standard curves and applied dilution coefficients. The limits of detection and quantification for certain phenolics are presented in Table 1.

The chromatograms of the HPLC separations are presented in the Supplementary Materials (Figures S1–S14).

In the case of all the chemical analyses performed within the study, three replications of each of the technological product types were tested.

### 2.3. Statistical Analyses

Statistical analyses were performed in the R statistical environment, version 4.4.1. [29] and included one-way analysis of variance (ANOVA), followed by the post-hoc Tukey’s HSD test (packages: “multcompView”, “ggpubr”, “broom” and “AICcmodavg”), with significance level set at the *p* < 0.05. Results were visualized using “hrbrthemes”, “ggplot2”, and “ggtext” packages in R. In addition, the Principal Component Analysis and k-means clustering was carried out and visualized using “devtools”, “ggplot2”, “ggrepel”, “ggfortify”, and “factoextra” R packages.

## 3. Results and Discussion

### 3.1. Dry Matter and Vitamin C

More and more consumers are conscious of the importance of nutrition in maintaining good health, which is why information about bioactive compounds is important to them in their shopping decisions. One of these compounds is vitamin C, which, along with β-carotene (provitamin A), vitamin A (retinol), and vitamin E (tocopherol), is known as an antioxidant vitamin [30]. As an exogenous compound for humans, vitamin C must be supplied in food, and the referenced daily intake of vitamin C is 80 mg [30]. According to the literature, Japanese quince fruits are a relatively rich source of vitamin C, containing from 55 to 243 mg per 100 g of fruit, depending on the variety (genotype) and environmental conditions [22]. According to other authors, vitamin C content in the fresh fruits of the most common quince varieties grown in Poland ranges from 68.8 to 159.3 mg/100 g [31]. For comparison, the vitamin C content in lemon juice is 30–70 mg per 100 g [8].

As expected, the processed products tested within this study were generally characterized by lower contents of vitamin C compared to the data previously reported for fresh fruits. Moreover, the studied quince product samples differed significantly in the vitamin C content (including DHA and L-Asc) depending on the processing (Table 2), with a general trend towards higher vitamin C concentrations in less processed products. When comparing the seven quince products, juice appeared to be about five times richer in vitamin C (34.48 mg/100 g fresh weight) compared to jam and syrups, which contained from 5.78 to 6.92 mg/100 g fresh weight of vitamin C (Table 2). High temperature is one of the contributing factors that causes significant losses of ascorbic acid in the production of fruit preserves. This was confirmed in research on strawberries, where the greatest losses of vitamin C in strawberry juice production were caused by pressing and pasteurization. The pressing process caused a loss of vitamin C of about 22%, while high-temperature pasteurization reduced its content by 35% compared to filtered juice. The lowest losses of vitamin C were observed during the processing of strawberries into purée (12%) compared to raw strawberries [32]. In fruit and vegetable processing, the initial thermal treatments can cause significant loss of water-soluble and oxygen-labile nutrients such as vitamin C. However, these nutrients are relatively stable during subsequent product storage, owing to the lack of oxygen [33].

The significant differences between the vitamin C content in products such as candied fruit, jams, and fruit in syrups in the present study could have been influenced by several factors. Using sugar in candied fruits can slow vitamin C degradation by reducing water activity and oxygen availability [34,35]. Candied fruits generally retain more vitamin C than, e.g., fried or boiled fruits, due to lower oxygen exposure and the protective effects of sugar. However, they lose more vitamin C than refrigerated fresh fruit due to heat treatment and extended storage durations [36]. In the case of jams, long-term exposure to high temperatures during processing leads to significant degradation of vitamin C, due to its thermal sensitivity [35,37]. Moreover, a prolonged process with the access of oxygen also accelerates the vitamin C oxidative degradation and loss [38,39]. In syrups, high temperatures also accelerate vitamin C breakdown via non-oxidative degradation mechanisms [35,36].

It is worth noting that no statistically significant differences in the content of vitamin C as well as DHA and L-Asc were observed between quince syrups depending on the sweeteners used (cane sugar, honey and xylitol). The vitamin C content in these products could have been impacted by the interplay of their chemical stability, pH levels, and the impact of thermal processing on ascorbic acid in the presence of these sweeteners [40,41,42,43]. Ascorbic acid is sensitive to pH levels, with higher stability at acidic pH values (pH 3–4). The type of sweetener used can influence the acidity of the preserves. In the case of xylitol as a sugar alcohol, the pH is neutral. Its use might result in a slight reduction in acidity, making ascorbic acid more vulnerable to degradation during heat treatment [44]. Moreover, xylitol lacks the antioxidant properties of honey and does not participate in the Maillard reaction which may produce by-products that stabilize ascorbic acid indirectly [45,46,47]. This might leave ascorbic acid more exposed to heat and oxygen, resulting in a higher degradation level. However, xylitol’s ability to reduce oxidation in certain food matrices could potentially help maintain the stability of vitamin C, which is sensitive to oxidation. Xylitol has also been shown to enhance the bioavailability of certain compounds, such as flavanols in green tea when combined with vitamin C. This suggests that xylitol might indirectly support the absorption or stability of vitamin C in specific formulations [48]. Also, the synergistic effect of sugar sweeteners and fruit compounds may result in lower degradation levels of vitamin C in preserves. Fruits contain enzymes like ascorbate oxidase that can degrade ascorbic acid. Honey and cane sugar might inhibit enzyme activity to some extent, while xylitol may not have the same effect, allowing for increased enzymatic degradation [45]. Honey has been shown to help retain moisture and reduce browning in products, which might indirectly help preserve vitamin C by maintaining a more stable environment [49].

In syrups, contrary to the other products tested, a lower content of the active form of vitamin C, L-Asc, than DHA was observed. The ratio of L-ascorbic acid to dehydroascorbic acid is a key indicator of vitamin C’s stability during processing. Several processing techniques can influence this ratio [50]. High temperatures accelerate the oxidation of L-ascorbic acid to DHA. Prolonged heating or higher temperatures may shift the ratio toward DHA or lead to the complete degradation of both forms [50,51]. Exposure to light and oxygen can also have an impact. Oxidative degradation is highly impacted by the presence of oxygen and light. Vacuum or inert-gas packaging can help maintain higher L-asc/DHA ratios [52,53].

There were statistically significant differences in the dry matter content between all the tested quince preserves, with dry matter content being generally higher in the products with a denser consistency (Table 2) [54].

### 3.2. Phenolic Compounds

Polyphenols are the main bioactive compounds in *Chaenomeles* [8]. The content of these compounds in fruit preserves is generally known to be affected by various processing techniques, such as cooking, dehydration, and other methods [55,56,57]. Thanks to technological processes, including high temperature, these and other fruit secondary metabolites can undergo isomerization, degradation, and polymerization reactions, creating new substances that usually have a weaker intensity of bitterness or astringency [58]. Thus, Japanese quince fruit processing is of great importance, showing the potential to shape the sensory properties of the fruit preserves.

The processed product samples tested in this study contained different levels of phenolic compounds. The quince juice was distinctly richer in polyphenols (sum) and phenolic acids than other preserves (Table 3). Syrup with xylitol was the second product with the highest content of these compounds. At the same time, no statistically significant differences were identified in the content of phenolic compounds (total) and, consequently, phenolic acids (total) and flavonoids (total) between the syrups sweetened with cane sugar and honey. The lowest polyphenol concentrations were detected in fruits in syrup and jam. When comparing phenolic acid content, quince juice and syrup with xylitol contained the highest amounts of these compounds, but in the case of flavonoids, the richest ones were candied fruits. At the same time, they turned out to be the third product in terms of polyphenol content, surpassing syrups based on cane sugar and honey as well as fruit in syrup and jam (Table 3). The concentrations of phenolic acids and flavonoids standardized to the dry substance of the tested quince products are presented in Supplementary Tables S1 and S2.

Heat treatment can degrade but also increase the content of some of the bioactive compounds in the final products. The increase can result from the destruction of the plant cell walls during processing and the extraction of polyphenols by breaking the chemical bonds of high molecular weight compounds. This results in the formation of soluble polyphenols with a lower molecular weight [59]. The antioxidant capacity of the final product is, however, not only attributed to the phenolics and organic acid content, but to the action of various compounds present in quince and to their possible synergistic and antagonistic effects.

The analysis of the results obtained for quince syrups suggests a potential effect of xylitol as a sweetener on the content of polyphenols, especially phenolic acids, but also flavonoids, in processed products. Rutkowska et al. [60] found that adding a polyphenol-rich extract and xylitol to muffins increased the content of phenolic acids and the antioxidant potential of the final product and even improved its safety in terms of the anisidine value. In another study on the effect of sugars (sucrose, as a reference, and fructose) and sugar alcohols (xylitol and erythritol) on the characteristics of blackberry jams, including the content of polyphenols, the authors confirmed that only xylitol resulted in a lower rate of polyphenols degradation [61]. Nowicka and Wojdyło previously reported that in a food matrix rich in phenolic compounds, the addition of xylitol had a protective effect on the content of polyphenols and their antioxidant activity [62]. The increase in phenolic compounds in processed fruit, as previously mentioned, may be due to the degradation of higher molecular weight phenolic compounds to smaller phenolic molecules. Furthermore, sugar derivatives formed in advanced stages of the Maillard reaction, which are not present in xylitol-containing (sugar-free) products, are known to accelerate the degradation of phenolic compounds [61]. As emphasized by Benedek et al. [61], many studies have shown that phenolic compounds do not follow a strictly defined reaction sequence. An increase in phenolic compounds was observed in some processed fruit products, while native phenolic compounds were degraded. The unpredictable fluctuations in these compounds’ contents were explained by many mechanisms. Sugar-derived products formed in advanced stages of the Maillard reaction (furfural and hydroxymethylfurfural), which are not present in sugar-free preserves, are known to accelerate the degradation of phenolic compounds [63,64,65]. Xylitol may exert protective effects on phenolic acids in fruit preserves also due to its interaction with water molecules, stabilizing phenolic acids by reducing water activity and thus inhibiting their hydrolytic degradation [66]. Additionally, xylitol’s thermal behavior can alter heat penetration during processing, potentially reducing excessive heat exposure of phenolic acids [67]. This could explain the observed differences in the content of total polyphenols, including flavonoids and phenolic acids, in the tested syrups produced with cane sugar, honey, and sugar alcohol–xylitol. When comparing the tested syrups, the highest contents of these compounds were found in the syrup sweetened with xylitol.

At the same time, some studies suggest that the effect of white sugar on phenolic concentration in fruit preserves is complex and can be influenced by factors such as sugar concentration, processing methods, and storage conditions. According to some literature, sugar used in preserves can act as a protective agent, potentially reducing the degradation of phenolic compounds during processing by creating a more stable environment [68].

As honey contains phenolic compounds, adding it to fruit preserves can increase their total phenolic content. At the same time, the phenolic compounds in honey may interact with those naturally present in fruits, potentially altering their overall concentration and activity in the preserves [69]. Our study had a limited focus, not considering all potential phenolic compounds, flavonoids, and other health-promoting secondary metabolites present in honey. However, this natural sweetener, known for its rich composition with well-documented medicinal properties (i.e., anti-inflammatory, antibacterial, antioxidant, and other biological activities), could be considered among strategies to improve the taste and the health-promoting properties of the quince fruit products [70].

**Figure 3:**
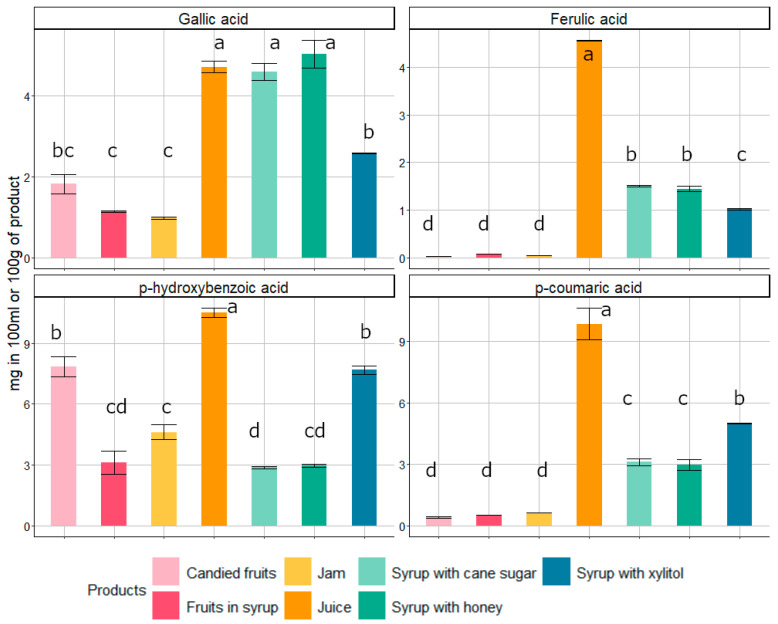
The concentration of identified phenolic acids (mg) in 100 g (candied fruits, fruits in syrup, jam) or 100 mL (pressed juice, syrups) of fresh weight of quince products studied (mean ± standard error). Different letters (a–d) above the bars indicate significant differences in the content of particular compounds between the products (Tukey’s HSD test, *p* < 0.001).

Phenolic acids constitute an integral part of the human diet, with many plant products, including quince preserves, being rich sources of *p*-hydroxybenzoic and *p*-coumaric acids [17]. Qualitative analysis of phenolic acids in the studied quince preserves revealed the following compounds: ferulic, gallic, *p*-coumaric, and *p*-hydroxybenzoic acids (Figure 3).

The tested products differed in the concentrations of phenolic acid. The contents of *p*-hydroxybenzoic acid were higher in comparison to other phenolic acids in most of the tested preserves, except for syrups. In both kinds of syrups, the predominant phenolic acid was gallic acid. Quince juice contained the highest amounts of all phenolic acids, which was especially noticeable for *p*-hydroxybenzoic acid (10.51 ± 0.22 mg/100 g fresh weight) and for *p*-coumaric acid (9.84 ± 0.77 mg/100 g fresh weight). In the candied fruits, fruits in syrup and jams, gallic acid was dominant, while in the other, less processed products, it was *p*-hydroxybenzoic acid (Figure 3). Home and industrial food processing methods and conditions are considered among the important factors influencing phenolic acid contents in final products [71]. The content of these compounds depends on the nature of the process, the duration of processing, and the initial composition of the raw material being processed [17,72].

**Figure 4:**
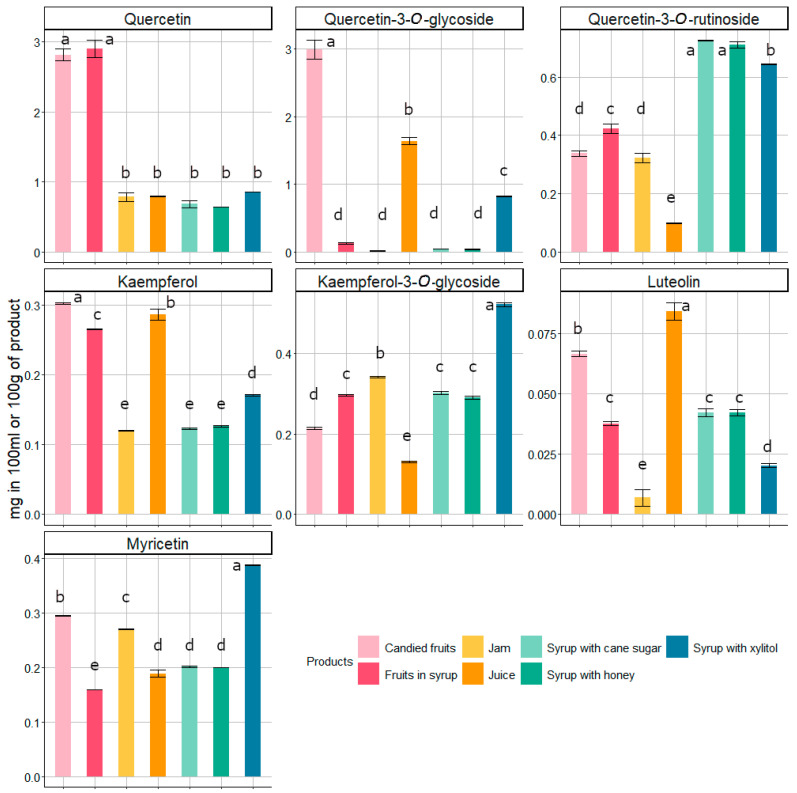
The concentration of flavonoids (mg) in 100 g (candied fruits, fruits in syrup, jam) or 100 mL (pressed juice, syrups) of fresh weight of quince products studied (mean ± standard error). Different letters (a–e) above the bars indicate significant differences in the content of particular compounds between the products (Tukey’s HSD test, *p* < 0.001).

The contents of flavonoids in the tested products were much lower than that of phenolic acids, as shown in Figure 4. Analysis of flavonoids allowed for the identification of seven compounds: kaempferol, kaempferol-3-*O*-glycoside, quercetin, quercetin-3-*O*-rutinoside, quercetin-3-*O*-glycoside, luteolin, and myricetin. Quercetin was the main flavonoid in the quince products, showing the highest concentration in candied fruits and fruits in syrup. At the same time, quercetin-3-*O*-rutinoside was the main flavonoid in all three syrups. The syrups with cane sugar and honey stood out significantly from other products in terms of quercetin-3-*O*-rutinoside content, while syrup with xylitol stood out in terms of kaempferol-3-*O*-glycoside content. Among the flavonoids that were detected in tested samples, luteolin was present in the smallest amounts (Figure 4). In most cases of highly processed products, glycoside forms of flavonoids were dominant (e.g., syrups), in contrast to pressed juice (the least processed product). This is related to the fact that flavonoids in plants can occur separately in the form of aglycones or in bound forms, e.g., as glycosides (with sugars). During technological processes, aglycone forms are created as a result of hydrolysis. The transformation depends on the properties of individual compounds [17], and their significant changes in structure may affect their biological activity [8]. Under the influence of temperature during processing, the level of free flavonols in the processed product increases due to hydrolysis and extraction from the food matrix, which also affects the change in the bioavailability of glycosylated forms and, consequently, their beneficial effect on the body [8].

### 3.3. Principal Component Analyses

**Figure 5:**
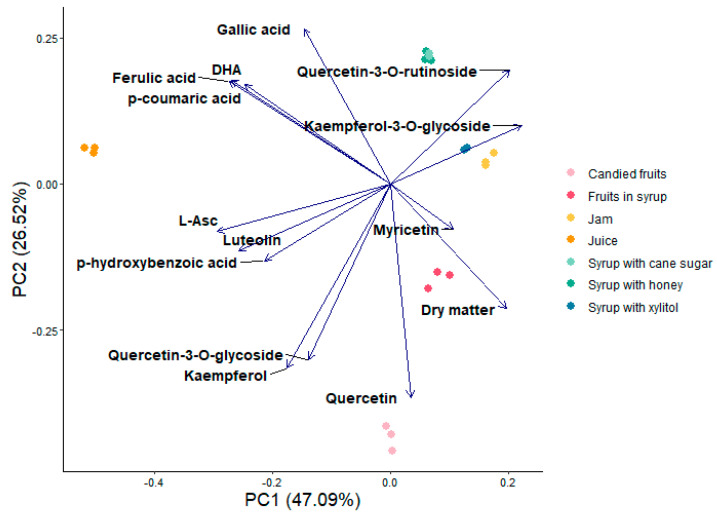
Principal component analysis of bioactive compounds concentrations in seven Japanese quince fruit products. PC1—1st principal component. PC2—2nd principal component.

**Figure 6:**
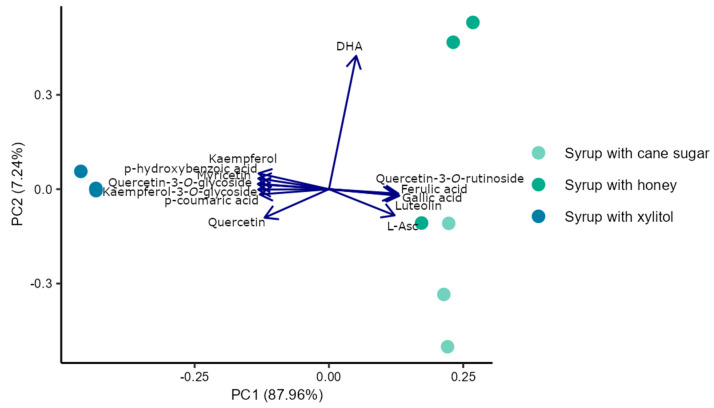
Principal component analysis of bioactive compounds in three Japanese quince fruit syrups produced with cane sugar, honey, and xylitol.

To objectify the diversity of bioactive compounds present in Japanese quince products and to explore the possible differences and similarities in the composition of products processed in different ways and products produced using different sweeteners (Figure 5 and Figure 6), a tool for data exploration in the form of principal component analysis (PCA) was used. The results of the analysis of samples of various products were presented on a two-dimensional graph of the first two components PC1 vs. PC2 (Figure 5). The first component explained over 47.1% of the variation, while the second explained over 26.5%. The analysis demonstrated that the processing method resulted in a clear separation of the results in five groups: (1) syrups with cane sugar and honey, (2) jam together with syrups with xylitol, (3) fruits in syrup, (4) candied fruits, (5) juice. The PCA plot also shows a positive association between the low level of processing (pressed juice) and concentrations of the majority of the tested bioactive compounds (except for quercetin-3-*O*-rutinoside, myricetin and kaempferol-3-*O*-glycoside concentration) (Figure 5).

By presenting the PCA results on a graph, it was possible to notice similarities and differences between the products tested, taking into account all the identified bioactive compounds. Among the more intensively processed products, the greatest differences can be noticed between candied fruits and syrups (with honey and cane sugar). The compounds characteristic for these products are quercetin and quercetin-3-*O*-rutinoside (for candied fruits and syrups, respectively). The products most similar to each other in terms of the content of the compounds tested were two types of syrups (with honey and cane sugar), and jam and syrup with xylitol.

**Figure A1:**
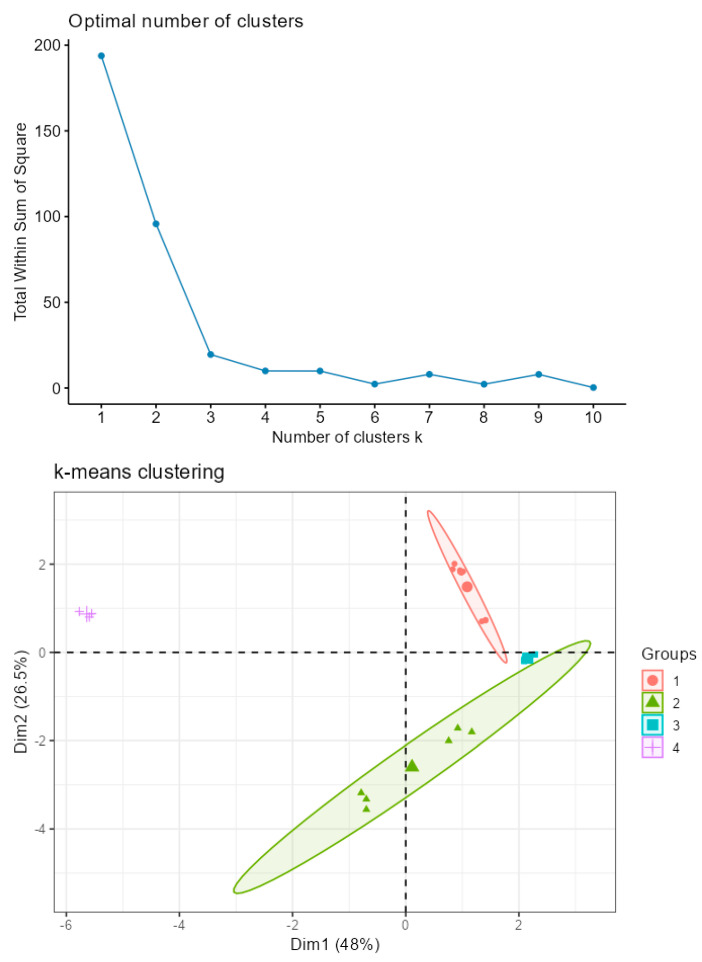
The result of k-means clustering presented as an optimal number of clusters as well as clusters. 1—1st cluster including syrups produced with honey, syrups produced with cane sugar and syrups produced with xylitol. 2—2nd cluster including candied fruits and fruits in syrup. 3—3rd cluster including quince jam. 4—4th cluster including quince juice. Dim1 and Dim2—dimensions 1 and 2. k—numbers of clusters.

Additionally, all samples were grouped using k-means clustering, which gave four clusters (Figure A1 in the Appendix A): candied fruits and fruits in syrup (first cluster), juice (second cluster), jam (third cluster), and all syrups together (fourth cluster). Therefore, principal component analysis was performed to explore further the possible differences and similarities in the composition of three syrups made with the addition of different sweeteners. The results were also presented in a two-dimensional graph of the first two components PC1 vs. PC2 (Figure 6). The first and second components explained 88% and 7% of variation, respectively. The PCA plot also shows a positive association of xylitol addition with the content of kaempferol, myricetin, quercetin, keampferole-3-*O*-glycoside, and quercetin-3-*O*-glycoside, as well as *p*-hydroxybenzoic acid and *p*-coumaric acid. Other polyphenols and L-Asc were positively associated with syrups produced with cane sugar or honey instead of xylitol. This analysis shows these compounds might differentiate products produced with different sweeteners. Based on these results, it can be assumed that there is a relationship between using xylitol as a sweetener and a higher content of most of the analyzed flavonoids and phenolic acids in the quince fruit syrups.

To summarize, the primary goal of this study was to investigate the concentrations of vitamin C and phenolic compounds in Japanese quince fruit preserves available on the market, to see whether the applied processing technologies allow the well-documented significant health-promoting compounds of quince fruit to be brought through the processing steps. We found this especially relevant since unprocessed (raw, fresh) Japanese quince fruits are characterized by organoleptic properties that face limited consumer acceptance. Thus, it could be of interest to consumers whether the processed Japanese quince products, which show much more attractive sensory attributes, also keep the health-promoting values of the raw material. Since the studied preserves were characterized by different processing levels/techniques/intensities, and different sweeteners were used in their production, we also decided to discuss the potential impact of these factors on the final product quality. Even though this analysis does not allow for the total elimination of other factors that could have impacted the observed differences between the studied preserves, it provides insights for future research aimed at determining whether different sweeteners and their mixtures, especially natural ones, can facilitate technological innovation without compromising the nutritional value and sensory acceptance of fruit products.

## 4. Conclusions

The study confirmed that the Japanese quince fruit preserves can be generally considered a rich source of vitamin C and selected phenolics. The obtained results also indicated a differentiation in terms of the analyzed compound concentrations among the studied products, produced with the involvement of diverse processing methods and sweeteners. Our results suggest that xylitol may be a good choice as a sugar substitute in heat-treated products. Further research directed toward optimizing thermal and non-thermal methods and toward the selection of relevant sweeteners that have the potential to retain valuable bioactive compounds in the final product would be of relevance.

The current study has limitations, but it provides an impetus for future research aimed at determining whether different sweeteners and their mixtures, especially natural ones, can facilitate technological innovation without compromising the nutritional value and sensory acceptance of fruit products. The different sugar alcohols, taking into account their specific characteristics, have great potential for use in these products.

Further studies should more closely investigate the stability of phenolic acids and other polyphenolic compounds during subsequent processing (i.e., heating and drying) and storage steps, uncovering degradation kinetics and reflecting percentage losses of these compounds over time or/and during specific treatments. Additionally, conducting laboratory simulation experiments using standard polyphenol and phenolic acid samples to mimic industrial production steps and subsequently determining their loss rates and stability could provide valuable insights into underlying mechanisms, supporting further optimization and scaling-up in industrial applications. In addition to the bioactive compound profiles targeted in this study, further studies should also investigate the antioxidant activity of quince fruit products, their sensory properties that determine consumer acceptance, and the bioaccessibility of their key health-promoting compounds. Moreover, studies on other sweeteners, including artificial ones, could be of interest, considering the trend towards using these agents as sugar replacements in many sectors of the food processing industry. This is particularly important in the face of the growing share of processed foods in overall diets, and the related health concerns and challenges.

## Figures and Tables

**Table 1:** The limits of detection (LOD) and quantification (LOQ) for phenolic compounds (mg in 100 g f.w.).

Compounds	mg in 100 g f.w.	
	LOD	LOQ
gallic acid	0.276	0.827
*p*-hydroxybenzoic acid	0.459	1.378
*p*-coumaric acid	0.147	0.441
ferulic acid	0.007	0.022
quercitin-3-*O*-glycoside	0.010	0.020
kaempferol-3-*O*-glycoside	0.037	0.110
myricetin	0.037	0.110
quercetin	0.092	0.276
quercetin-3-*O*-rutinoside	0.018	0.055
luteolin	0.002	0.006
kaempferol	0.037	0.110

LOD—limit of detection; LOQ—limit of quantification.

**Table 2:** The content of dry matter (g) and concentration of vitamin C (mg) in 100 g (candied fruits, fruits in syrup, jam) or 100 mL (pressed juice, syrups) of fresh weight of quince products studied (mean ± standard error).

Quince Products	Dry Matter	Vitamin C (DHA + L-Asc)	DHA	L-Asc
Candied fruits	70.83 ± 0.59 a	11.27 ± 0.03 b	1.91 ± 0.04 d	9.36 ± 0.01 b
Fruits in syrup	37.52 ± 1.22 d	11.70 ± 0.24 b	2.46 ± 0.24 c	9.24 ± 0.01 b
Jam	33.45 ± 0.10 e	5.78 ± 0.05 c	2.59 ± 0.04 c	3.19 ± 0.01 c
Pressed juice	6.80 ± 0.04 f	34.48 ± 0.99 a	8.22 ± 0.09 a	26.26 ± 0.91 a
Syrup with cane sugar	37.83 ± 0.99 d	6.95 ± 0.02 c	3.79 ± 0.02 b	3.16 ± 0.00 c
Syrup with honey	43.20 ± 0.03 c	6.95 ± 0.08 c	3.89 ± 0.04 b	3.05 ± 0.04 c
Syrup with xylitol	53.74 ± 0.11 b	6.62 ± 0.04 c	3.80 ± 0.00 b	2.82 ± 0.04 c
ANOVA *p*-values	<0.001	<0.001	<0.001	<0.001

Values in the same column followed by different letters (a–f) are significantly different at the 5% level of probability (based on Tukey’s HSD test). DHA—dehydroascorbic acid. L-Asc—L-ascorbic acid.

**Table 3:** The concentration of phenolic acids, flavonoids and their sum (mg) in 100 g (candied fruits, fruits in syrup, jam) or 100 mL (pressed juice, syrups) of fresh weight of quince products studied (mean ± standard error).

Quince Products	Polyphenols (Phenolic Acids + Flavonoids)	Phenolic Acids (sum)	Flavonoids (sum)
Candied fruits	17.14 ± 0.53 c	10.11 ± 0.68 c	7.03 ± 0.23 a
Fruits in syrup	9.09 ± 0.65 e	4.88 ± 0.58 d	4.21 ± 0.12 b
Jam	8.17 ± 0.46 e	6.31 ± 0.39 d	1.86 ± 0.08 d
Pressed juice	32.84 ± 0.98 a	29.61 ± 0.93 a	3.22 ± 0.06 c
Syrup with cane sugar	14.21 ± 0.07 d	12.09 ± 0.02 c	2.12 ± 0.05 d
Syrup with honey	14.48 ± 0.25 d	12.43 ± 0.26 c	2.05 ± 0.01 d
Syrup with xylitol	19.71 ± 0.21 b	16.28 ± 0.22 b	3.43 ± 0.01 c
ANOVA *p*-values	<0.001	<0.001	<0.001

Values in the same column followed by different letters (a–e) are significantly different at the 5% level of probability (based on Tukey’s HSD test).

## Data Availability

Data will be made available upon request by the corresponding author.

## Supplementary Materials

The following supporting information can be downloaded at: https://www.mdpi.com/article/10.3390/foods14081369/s1, Supplementary Figures S1–S14: The chromatograms of the HPLC separations; Supplementary Tables S1 and S2: The concentration of phenolics (mg) in 100 g of dry weight of quince products studied.
