# Supplementary material for: The Concentrations of Phenolic Compounds and Vitamin C in Japanese Quince (Chaenomeles japonica) Preserves
Source: Foods. 2025 Apr 16;14(8):1369. doi: 10.3390/foods14081369 (PMC12027240; doi:10.3390/foods14081369)
Supplement: Supplementary file 1 [file foods-14-01369-s001.zip › foods-3571028-supplementary/foods-3571028-Supplementary Materials-Figures.pdf]

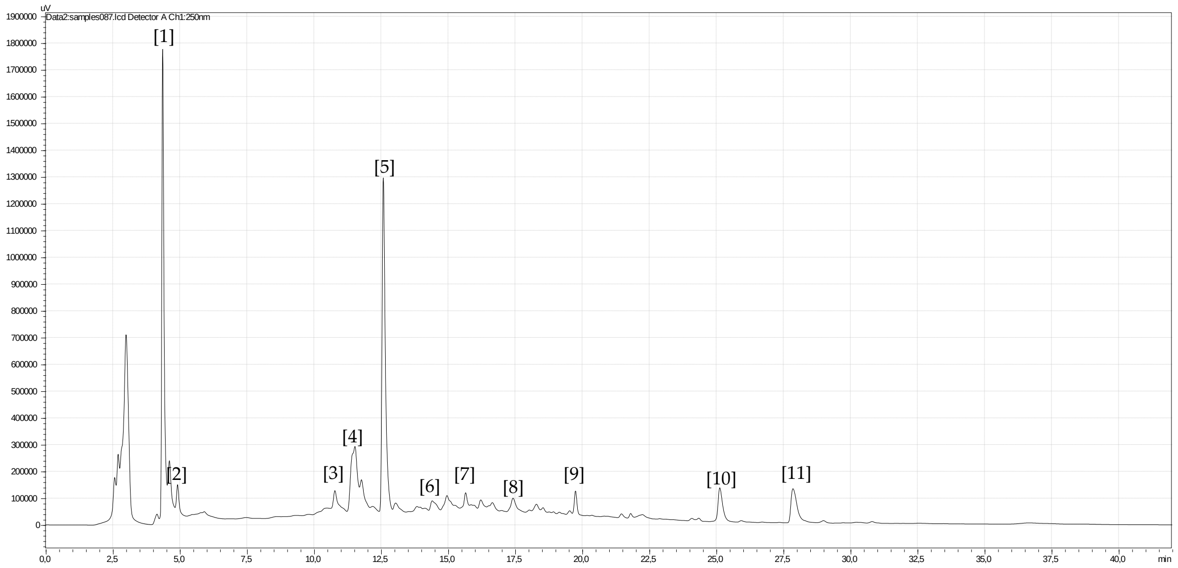

Figure S1. The example of chromatogram of identified phenolic compounds in candied fruits: (1) gallic acid, (2) p-hydrobenzoic acid, (3) p-coumaric acid, (4) ferulic acid, (5) quercetin-3-O-rutinoside, (6) kaempferol-3-O-glucoside, (7) myricetin, (8) quercetin, (9) luteolin, (10) kaempferol, (11) quercetin-3-O-glucoside.

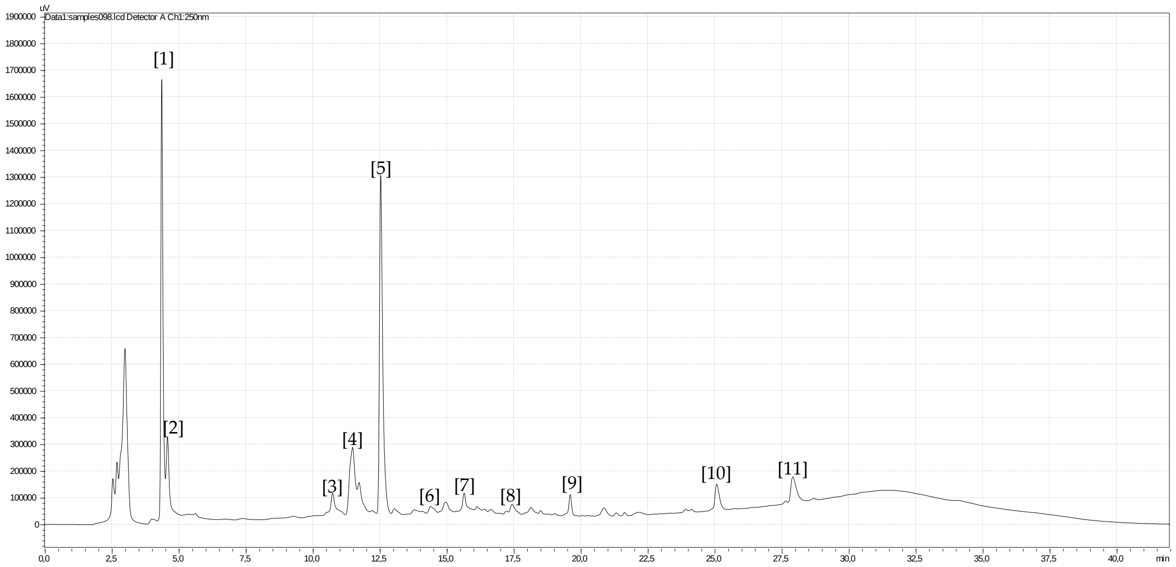

Figure S2. The example of chromatogram of identified phenolic compounds in fruits in syrup: (1) gallic acid, (2) p-hydrobenzoic acid, (3) p-coumaric acid, (4) ferulic acid, (5) quercetin-3-O-rutinoside, (6) kaempferol-3-O-glucoside, (7) myricetin, (8) quercetin, (9) luteolin, (10) kaempferol, (11) quercetin-3-O-glucoside.

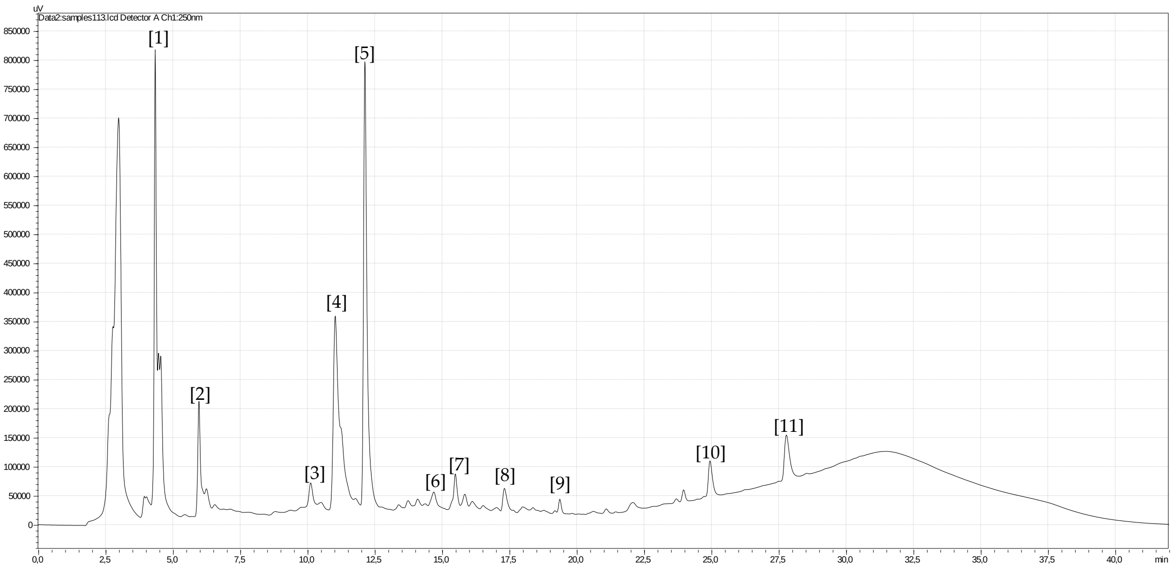

Figure S3. The example of chromatogram of identified phenolic compounds in jam: (1) gallic acid, (2) p-hydrobenzoic acid, (3) p-coumaric acid, (4) ferulic acid, (5) quercetin-3-O-rutinoside, (6) kaempferol-3-O-glucoside, (7) myricetin, (8) quercetin, (9) luteolin, (10) kaempferol, (11) quercetin-3-O-glucoside.

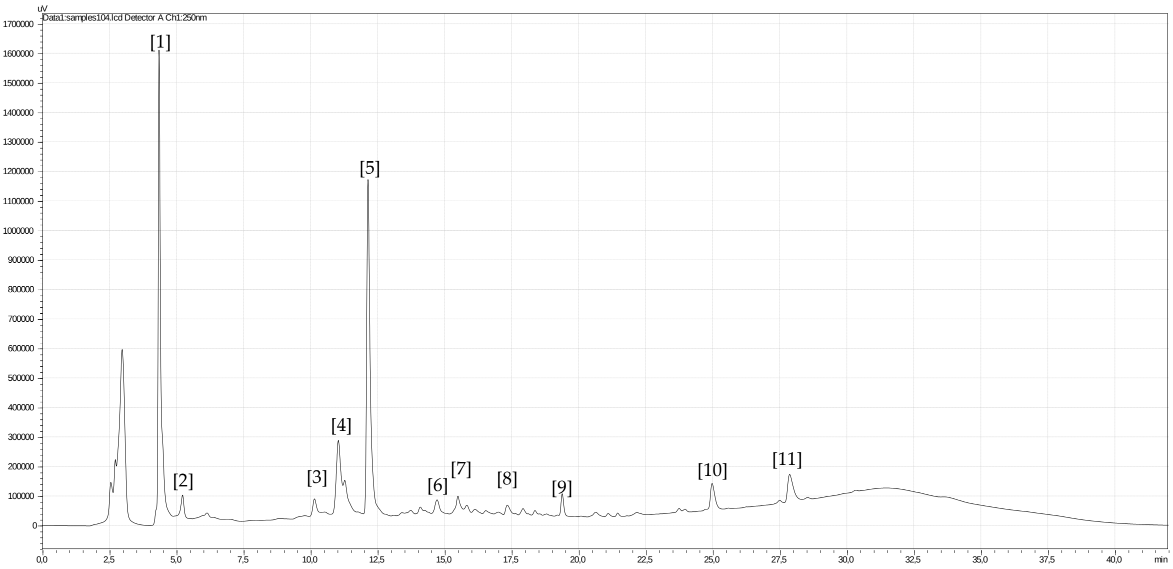

Figure S4. The example of chromatogram of identified phenolic compounds in pressed juice: (1) gallic acid, (2) p-hydrobenzoic acid, (3) p-coumaric acid, (4) ferulic acid, (5) quercetin-3-O-rutinoside, (6) kaempferol-3-O-glucoside, (7) myricetin, (8) quercetin, (9) luteolin, (10) kaempferol, (11) quercetin-3-O-glucoside.

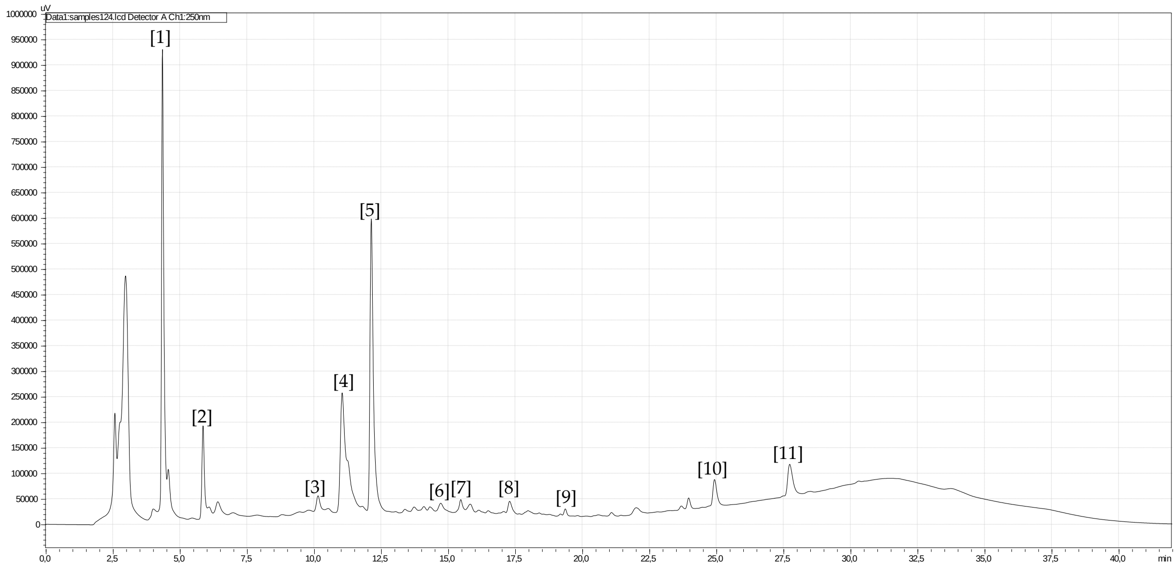

Figure S5. The example of chromatogram of identified phenolic compounds in syrup with cane sugar: (1) gallic acid, (2) p-hydrobenzoic acid, (3) p-coumaric acid, (4) ferulic acid, (5) quercetin-3-O-rutinoside, (6) kaempferol-3-O-glucoside, (7) myricetin, (8) quercetin, (9) luteolin, (10) kaempferol, (11) quercetin-3-O-glucoside.

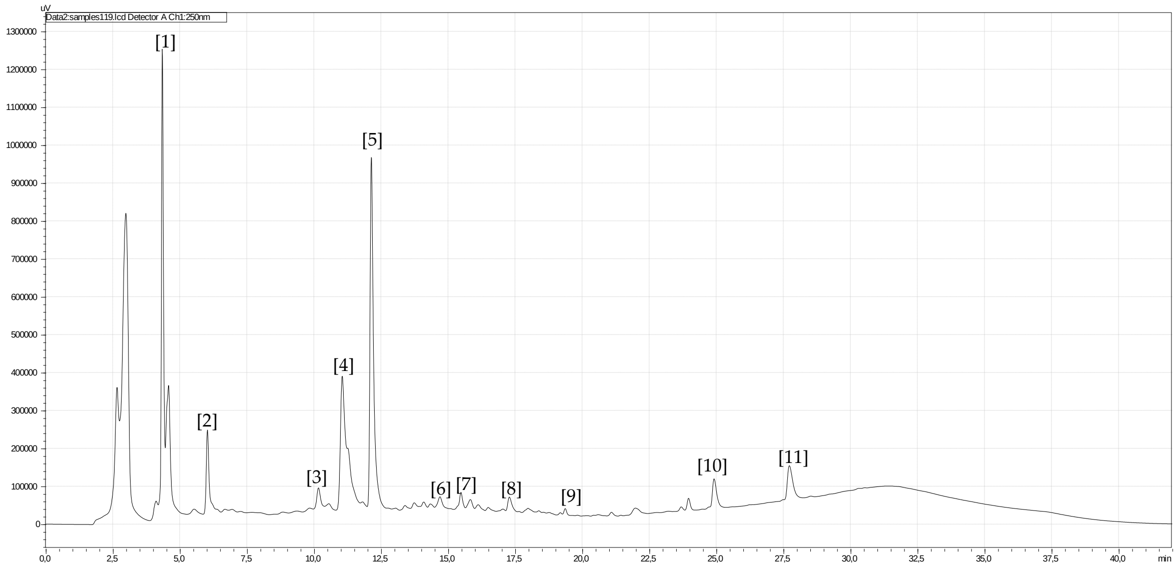

Figure S6. The example of chromatogram of identified phenolic compounds in syrup with honey: (1) gallic acid, (2) p-hydrobenzoic acid, (3) p-coumaric acid, (4) ferulic acid, (5) quercetin-3-O-rutinoside, (6) kaempferol-3-O-glucoside, (7) myricetin, (8) quercetin, (9) luteolin, (10) kaempferol, (11) quercetin-3-O-glucoside.

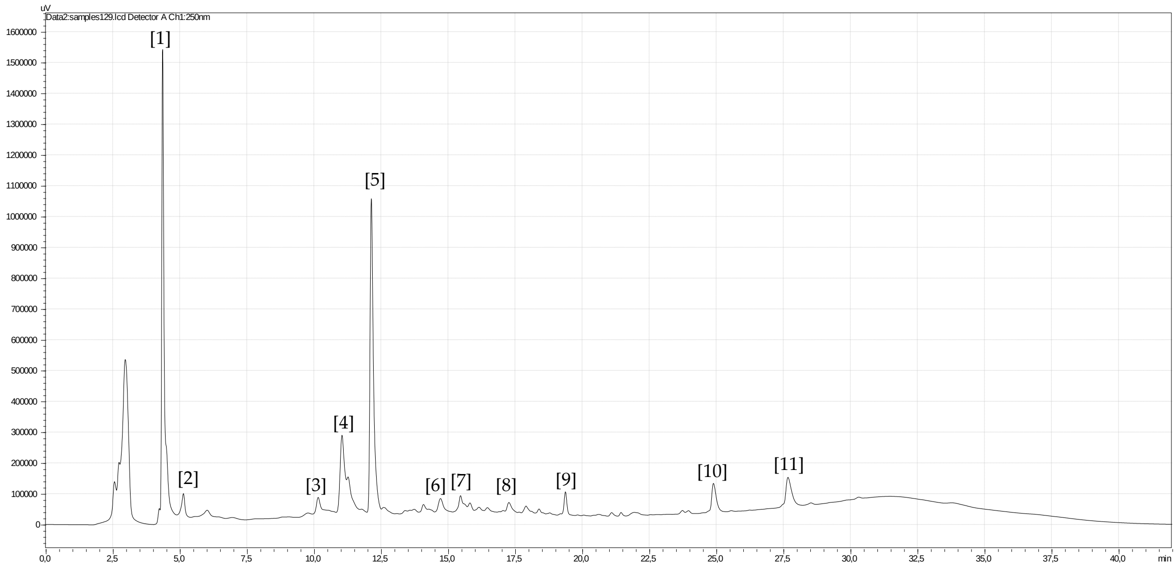

Figure S7. The example of chromatogram of identified phenolic compounds in syrup with xylitol: (1) gallic acid, (2) p-hydrobenzoic acid, (3) p-coumaric acid, (4) ferulic acid, (5) quercetin-3-O-rutinoside, (6) kaempferol-3-O-glucoside, (7) myricetin, (8) quercetin, (9) luteolin, (10) kaempferol, (11) quercetin-3-O-glucoside.

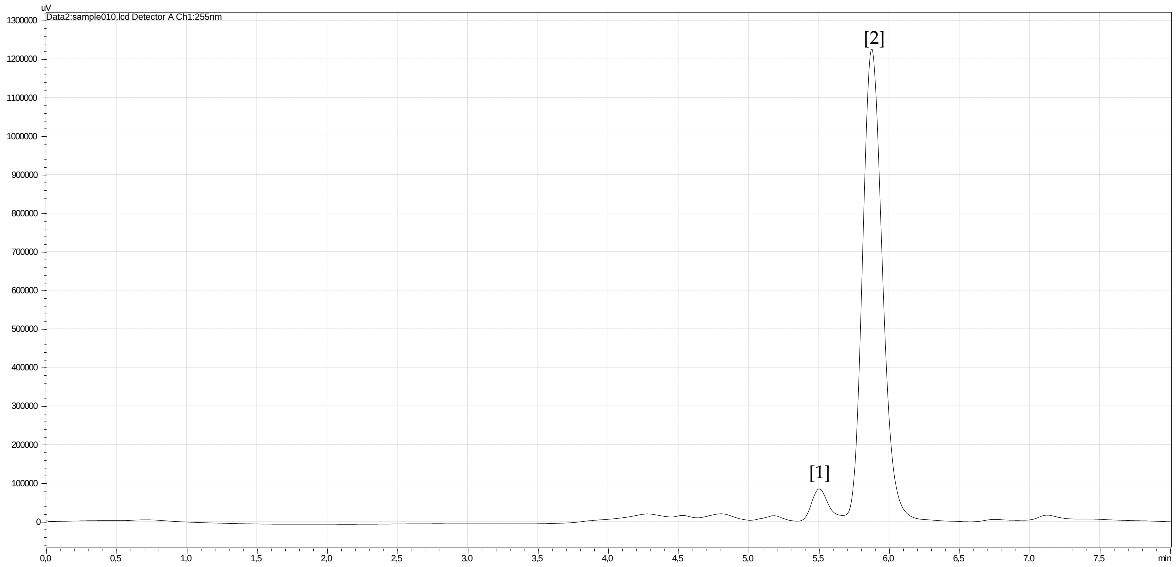

Figure S8. The example of chromatogram of identified vitamin C compounds in candied fruits: (1) dehydroascorbic acid, (2) l-ascorbic acid.

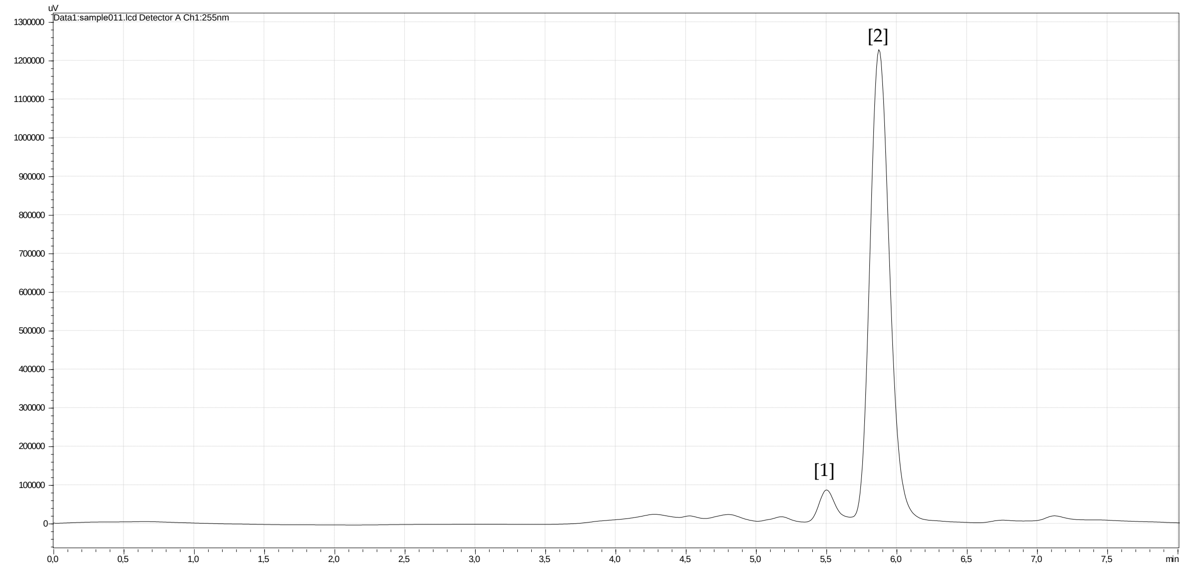

Figure S9. The example of chromatogram of identified vitamin C compounds in fruits in syrup: (1) dehydroascorbic acid, (2) l-ascorbic acid.

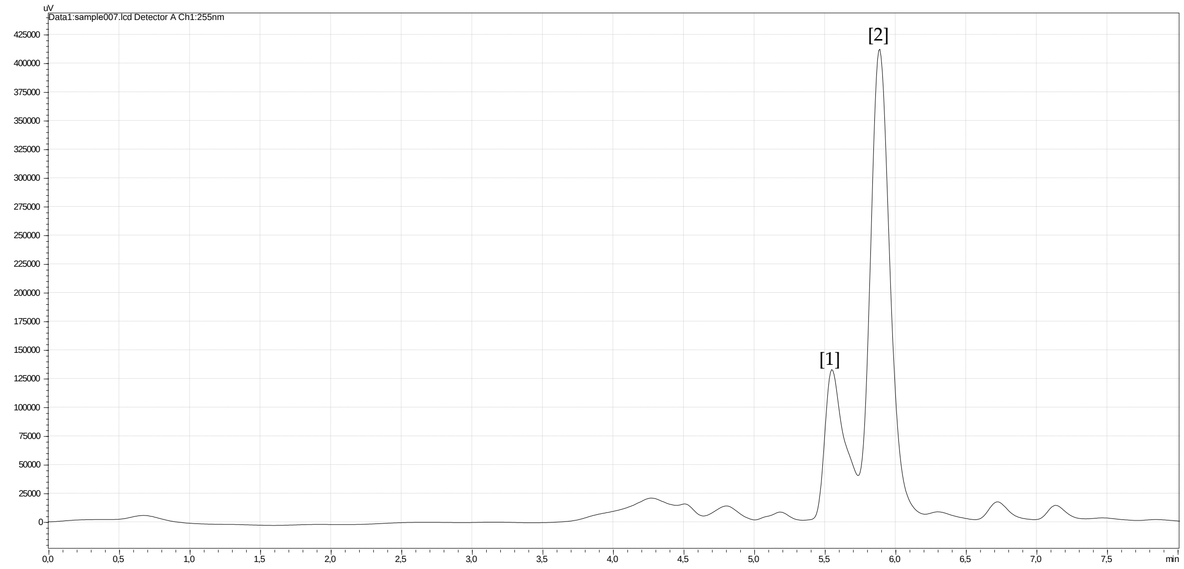

Figure S10. The example of chromatogram of identified vitamin C compounds in jam: (1) dehydroascorbic acid, (2) l-ascorbic acid.

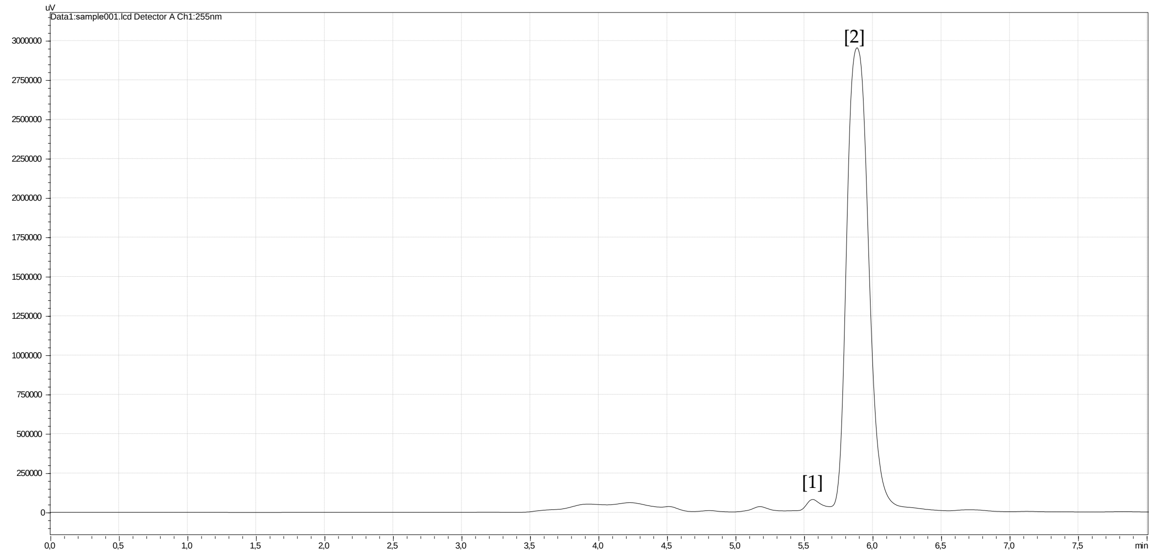

Figure S11. The example of chromatogram of identified vitamin C compounds in pressed juice: (1) dehydroascorbic acid, (2) l-ascorbic acid.

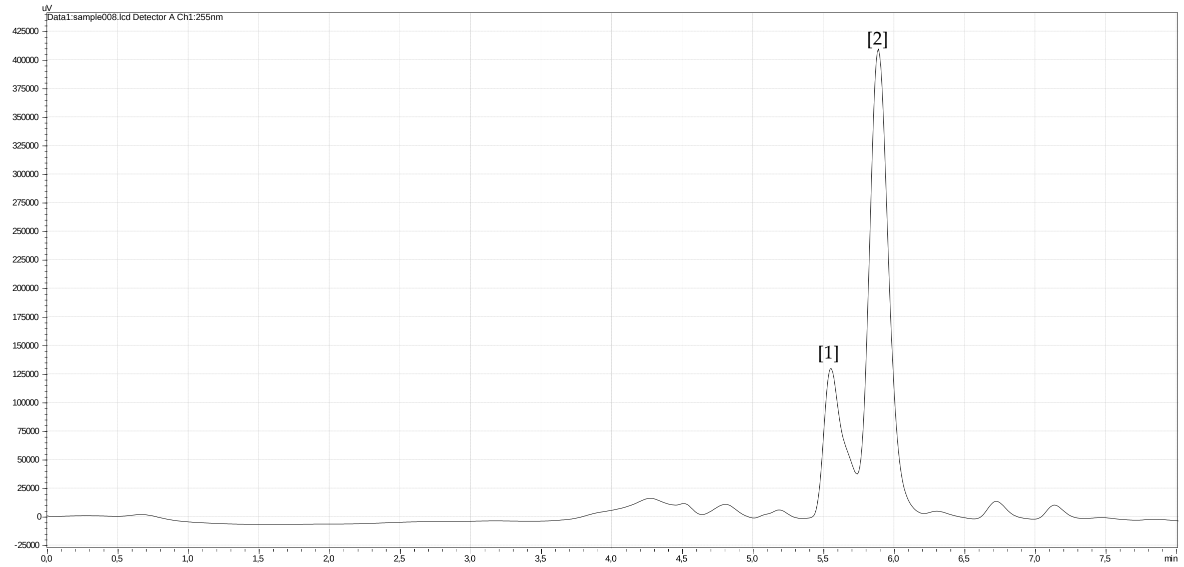

Figure S12. The example of chromatogram of identified vitamin C compounds in syrup in cane sugar: (1) dehydroascorbic acid, (2) l-ascorbic acid.

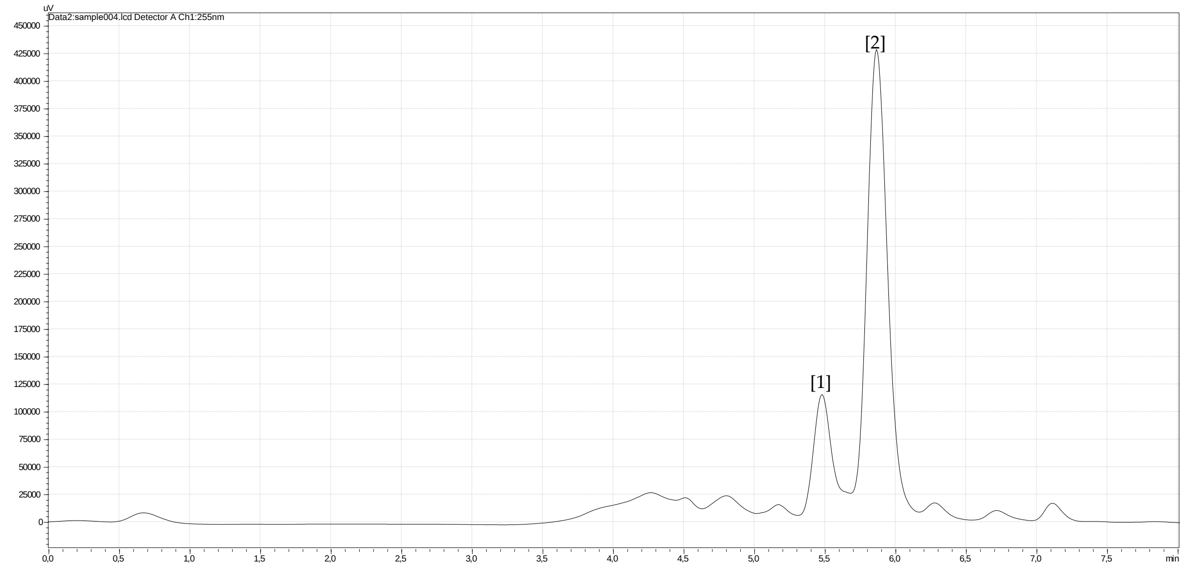

Figure S13. The example of chromatogram of identified vitamin C compounds in syrup with honey: (1) dehydroascorbic acid, (2) l-ascorbic acid.

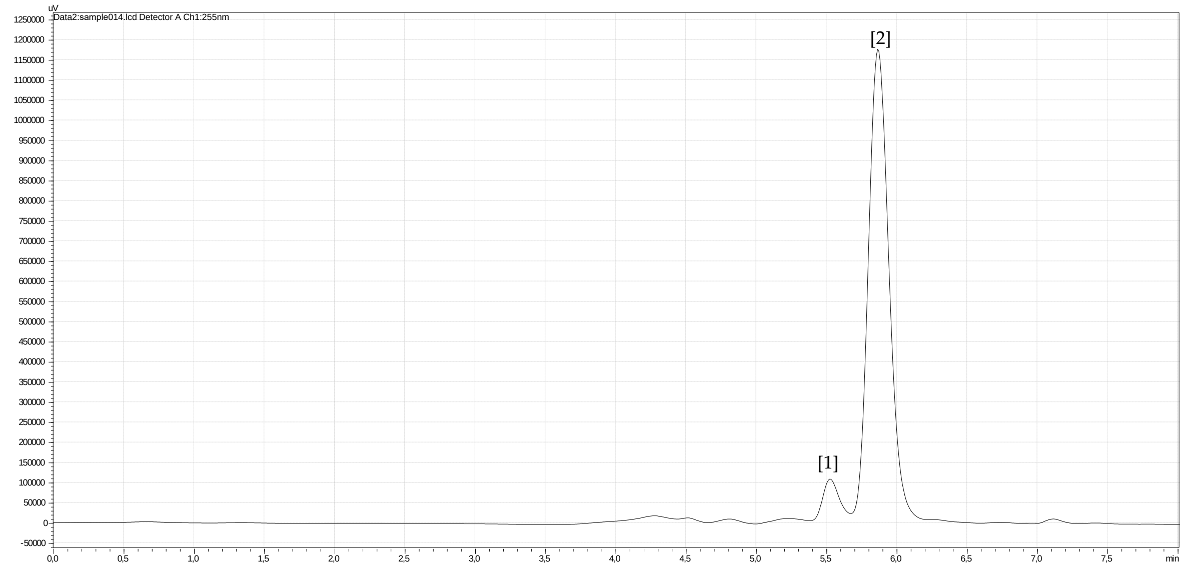

Figure S14. The example of chromatogram of identified vitamin C compounds in syrup with xylitol: (1) dehydroascorbic acid, (2) l-ascorbic acid.
