# Supplementary material for: The Concentrations of Phenolic Compounds and Vitamin C in Japanese Quince (Chaenomeles japonica) Preserves
Source: Foods. 2025 Apr 16;14(8):1369. doi: 10.3390/foods14081369 (PMC12027240; doi:10.3390/foods14081369)
Supplement: Supplementary file 1 [file foods-14-01369-s001.zip › foods-3571028-supplementary/foods-3571028-Supplementary Materials-Tables.pdf]

Table S1. The concentration of phenolic acids (mg) in 100 g of dry weight of quince products studied (mean ± standard error).

Quince products	Gallic acid	Ferulic acid	*p*-coumaric acid	*p*-hydroxybenzoic acid
Candied fruits	2.57 ± 0.33 c	0.04 ± 0.00 e	0.6 ± 0.06 b	11.07 ± 0.69 bc
Fruits in syrup	3.02 ± 0.06 c	0.2 ± 0.00 e	1.46 ± 0.00 b	8.33 ± 1.57 bc
Jam	2.93 ± 0.07 c	0.14 ± 0.01 e	1.95 ± 0.02 b	13.83 ± 1.12 bc
Pressed juice	69.19 ± 2.03 a	67.04 ± 0.1 a	144.65 ± 11.31 a	154.55 ± 3.20 a
Syrup with cane sugar	10.1 ± 0.46 b	3.31 ± 0.05 b	6.88 ± 0.37 b	6.34 ± 0.11 bc
Syrup with honey	9.7 ± 0.66 b	2.79 ± 0.12 c	5.76 ± 0.51 b	5.74 ± 0.16 c
Syrup with xylitol	4.00 ± 0.02 c	1.59 ± 0.03 d	7.74 ± 0.04 b	11.92 ± 0.32 b
ANOVA *p*-values	<0.001	<0.001	<0.001	<0.001

Values in the same column followed by different letters (a – e) are significantly different at the 5% level of probability (based on Tukey's HSD test).

Table S2. The concentration of flavonoids (mg) in 100 g of dry weight of quince products studied (mean ± standard error).

Quince products	Kaempferol-3-*O*-glycoside	Quercetin-3-*O*-glycoside	Quercetin -3-O-rutinoside	Kaempferol	Luteolin	Myricetin	Quercetin
Candied fruits	0.30 ± 0.00 e	4.23 ± 0.19 b	0.48 ± 0.01 f	0.43 ± 0.00 c	0.09 ± 0.00 b	0.42 ± 0.00 c	3.98 ± 0.12 c
Fruits in syrup	0.79 ± 0.00 c	0.33 ± 0.03 cd	1.13 ± 0.04 d	0.71 ± 0.00 b	0.10 ± 0.00 b	0.42 ± 0.00 c	7.73 ± 0.33 b
Jam	1.02 ± 0.01 b	0.05 ± 0.01 d	0.96 ± 0.05 e	0.36 ± 0.00 c	0.02 ± 0.01 b	0.81 ± 0.00 b	2.35 ± 0.18 d
Pressed juice	1.93 ± 0.03 a	24.1 ± 0.77 a	1.45 ± 0.03 c	4.20 ± 0.12 a	1.24 ± 0.05 a	2.78 ± 0.10 a	11.67 ± 0.13 a
Syrup with cane sugar	0.67 ± 0.00 c	0.09 ± 0.00 cd	1.59 ± 0.00 a	0.27 ± 0.00 c	0.09 ± 0.00 b	0.44 ± 0.00 c	1.50 ± 0.11 de
Syrup with honey	0.56 ± 0.01 d	0.08 ± 0.01 d	1.37 ± 0.02 b	0.24 ± 0.00 c	0.08 ± 0.00 b	0.39 ± 0.00 c	1.24 ± 0.00 e
Syrup with xylitol	0.81 ± 0.01 b	1.28 ± 0.01 c	1.00 ± 0.00 d	0.26 ± 0.00 c	0.03 ± 0.00 b	0.60 ± 0.00 b	1.33 ± 0.00 de
ANOVA *p*-values	<0.001	<0.001	<0.001	<0.001	<0.001	<0.001	<0.001

Values in the same column followed by different letters (a – e) are significantly different at the 5% level of probability (based on Tukey's HSD test).
